# Assessment of Morphological and Functional Changes in Organs of Rats after Intramuscular Introduction of Iron Nanoparticles and Their Agglomerates

**DOI:** 10.1155/2015/243173

**Published:** 2015-02-19

**Authors:** Elena Sizova, Sergey Miroshnikov, Elena Yausheva, Valentina Polyakova

**Affiliations:** ^1^Orenburg State University, Prospekt Pobedy 13, Orenburg 460018, Russia; ^2^State Educational Institution, All-Russian Research Institute of Beef Cattle Breeding, RAAS, No. 29, 9 Yanvarya Street, Orenburg 460018, Russia; ^3^Orenburg State Medical Academy of Federal Agency in Public Health and Social Development, 6 Sovetskaya Street, Orenburg 460000, Russia

## Abstract

The research was performed on male Wistar rats based on assumptions that new microelement preparations containing metal nanoparticles and their agglomerates had potential. Morphological and functional changes in tissues in the injection site and dynamics of chemical element metabolism (25 indicators) in body were assessed after repeated intramuscular injections (total, 7) with preparation containing agglomerate of iron nanoparticles. As a result, iron depot was formed in myosymplasts of injection sites. The quantity of muscle fibers having positive Perls' stain increased with increasing number of injections. However, the concentration of the most chemical elements and iron significantly decreased in the whole skeletal muscle system (injection sites are not included). Consequently, it increased up to the control level after the sixth and the seventh injections. Among the studied organs (liver, kidneys, and spleen), Caspase-3 expression was revealed only in spleen. The expression had a direct dependence on the number of injections. Processes of iron elimination from preparation containing nanoparticles and their agglomerates had different intensity.

## 1. Introduction

Regular evolution of microelement preparations from nonorganic forms like salts oxides and so forth to organic derivatives is determined by lower toxicity and higher bioavailability in the latter [1].

At the same time, we are witnessing nanotechnologies developing and it becomes obvious that in the nearest future substances containing nanoparticles of elementary metals and their derivatives might offer an alternative to both organic and nonorganic compounds of microelements [2, 3]. This is due to lower toxicity in nanomaterials; in particular, nanosubstance of elementary selenium has proven to be less toxic if compared to selenomethionine. The experiment demonstrates a much lower level of toxicity in nanoselenium if compared to natriumselenit [4].

Nanocrystalline iron and a number of other substances taken in biotic dosages accelerate animal growth, enhance liver regeneration after partial hepatectomy, and facilitate tissue repair [5, 6].

These and other characteristics make the whole issue a relevant one. Finding out new effects of nanotype substances seems also important.

So, nanoparticles used as a source of microelements without clear toxic effects may come along with an increase of apoptosis in target organs [7].

High penetrability of metal nanoparticles in general and iron, in particular, may at intramuscular injections significantly increase the concentration of the introduced metal in blood or other body environments, which can lead to a variety of pathological changes [8, 9]. In this regard, studies aimed at developing solutions reducing the intensity of iron elimination in blood at intramuscular injections may be prospective. That can be achieved through the use of nanoparticle agglomerates.

The purpose of this work is to study the structural and functional reorganization of organs and tissues of rats and peculiarities of metabolism by intramuscular injection of iron nanoparticles and their agglomerates.

## 2. Materials and Methods

In the first experiment, the structural and functional reorganization of organs and tissues of animals at multiple injections of iron nanoparticle agglomerates was assessed (*d* = 431 ± 13.8 nm). The research was conducted on male Wistar rats (*n* = 15), 150–180 g, that received common ration in vivarium, once a week (for 12 weeks, with a total of 12 injections). The animals once a week were injected with iron nanoparticle agglomerates in femoral group of muscles in dosage of 2.0 mg/kg of weight. The injection sites were chosen at distances, and, in view of the muscle regeneration terms and respective recommendations [10], repeated injection in the same area was given no earlier than 3 weeks after.

In the second experiment, a comparative assessment of dynamics of iron supply into blood after intramuscular introduction of iron nanoparticles and their agglomerates was given taking into account the previously obtained data. Male Wistar rats weighing 170–200 g of the I group (*n* = 15) were injected with lyosols of iron nanoparticles (*d* = 80 ± 5 nm) in femoral muscle, of II group with agglomerates of iron nanoparticles (*d* = 431 ± 13.8 nm), and of III group with isotonic aqueous solution. Nanoparticle preparations were administered at a dose of 2.0 mg/kg of animal weight.

The experimental research on animals was done following the instructions set by the respective Russian Regulations (1987) and The Guide for the Care and Use of Laboratory Animals (National Academy Press Washington, DC, 1996). Iron nanoparticles were obtained through the method of high temperature condensation with a following modification by oxygen using a Migen-3 device [11]. The average size of the copper particles was 80 ± 5 nm; their surface oxidized to Fe_3_O_4_, with a nucleus of Fe^0^. The nanoparticle agglomerates substance was prepared in an isotonic solution using ultrasonic disperser (f, 35 kHz, N, 300 W, A, 10 mkA) in the following mode: thrice-repeated dispersion for 1 min each, recess for 3 min. Iron nanoparticle preparation was prepared by dispersion in isotonic solution within 30 minutes. Size of nanoparticles and their agglomerates was determined using electronic microscope JSM-740 IF. The nanoparticle concentration in the isotonic solution was 2 mg/mL. The animals were slaughtered through decapitation under Nembutal anesthesia within the following scheme: 1 day later, 3 days later, and 7 days later after each injection. The animals in the control group were injected with isotonic solution; slaughtering was carried out within the same terms. Three animals were used at each time point (both in the experimental group and in control).

For light microscopy, pieces of muscle tissue were fixed in a 10% neutral formalin solution. Paraffin sections (5-6 *μ*m) were stained with Mayer's hematoxylin and eosin. The copper nanoparticles in the parts under study were detected with the method of muriatic benzidine and ammonium thiocyanate, the presence of iron being detected through a specific reaction, Perls' reaction [12]. Immunohistochemical researches were carried out on paraffin slices with the use of monoclonal antibodies and imaging system “Bio Genex Super Sensytive Detection System” (USA) according to the records of the producer. Identify cell readiness to apoptosis, Caspase-3 expression.

Morphometric characteristics of nanoparticles and their complexes were observed by contact atomic force microscopy using multiviewing microscope SMM-2000 (JSC “PROTON-MIET Plant,” Russia).

Mineral content of the sample tissues (25 elements) was analyzed in the test laboratory of ANO “Centre for Biotic Medicine,” Moscow, Russia (accreditation certificate ROSS RU.0001.22ПЯ05 by Russian Federal Agency for Technique Regulation and Metrology, ISO 9001:2008 certificate 54Q10077 by Global Certification Ltd.).

The statistical processing of the obtained data was performed using the software package “Statistica 5” and “MS Excel.”

## 3. Results

The results of the first experiment show that iron depot is formed by intramuscular injection of iron nanoparticle agglomerates; it can be observed within 21 days.

**Figure 1:**
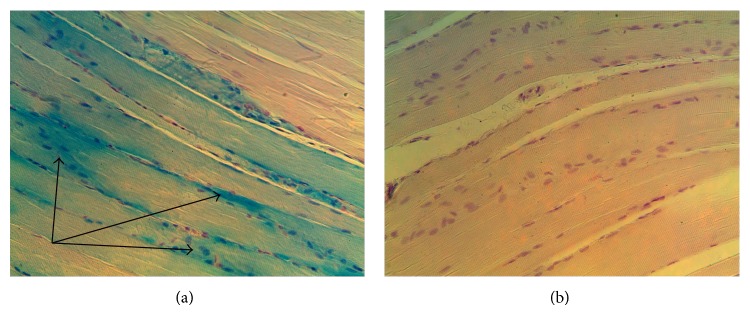
Injection site. (a) Experiment. The first day after a single injection of iron nanoparticles. Perls' reaction. (b) Control. Magn. 300. In the injection site, myosymplasts in comparison with adjacent fibers are loaded with iron; they have blue and green staining, indicated with arrows (Perls' reaction) (a); control has no such staining (b).

An inspection of the site of iron introduction on the first day after the first injection showed that the myosymplasts on that site were loaded with iron if compared to the adjacent fibers (Figure 1(a)) and compared to the control (Figure 1(b)), which was revealed with the Perls' reaction. On the third day, the injection site had remaining fibers loaded with iron, though their number decreased. Positive Perls' reaction was observed for endomysium elements.

**Figure 2:**
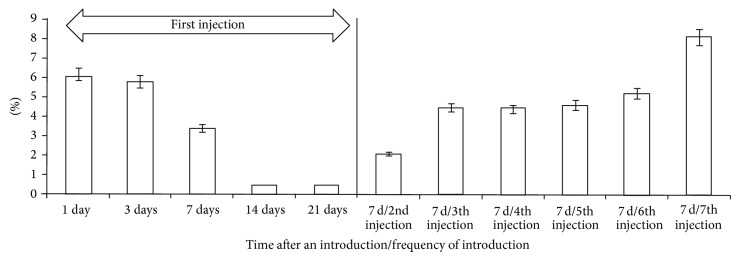
Number of muscle fibers with positive staining, Perls' reaction, %.

On the seventh day, the iron content in myosymplasts decreased in the injection site. Iron was predominantly found in connective-tissue macrophages of endo- and perimysium (Figure 2). Endo- and perimysium produced positive Perls' reaction at the injection site on days 14 and 21 after a single injection of nanoparticles.

**Figure 3:**
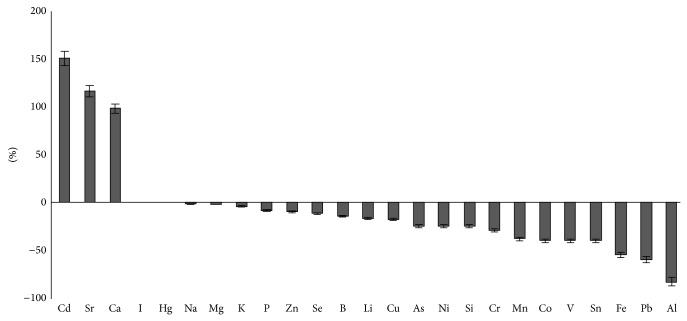
Difference in the concentration of chemical elements in muscle tissue (except injection site) of the experimental group in relation to the control group a day after the first injection with iron nanoparticle agglomerates, %.

At the same time a single introduction of iron agglomerates to skeletal muscles (muscles in injection site are not considered) was followed by a decrease in concentration of the most elements as early as on the first day. Iron concentration decreased by 54.8% (*P* < 0.01) (Figure 3).

**Figure 4:**
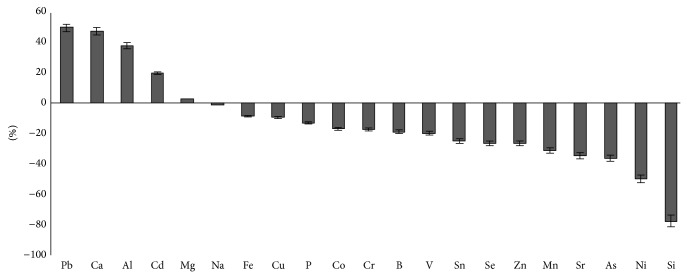
Difference in the concentration of chemical elements in muscle tissue (except injection site) of the experimental group in relation to the control group on day 21 after the first injection with iron nanoparticle agglomerates, %.

In the subsequent period, iron concentration became stable in skeletal muscles of experimental animals with small fluctuations. It exceeded the control level by 7.2% (*P* < 0.01) in seven days and gradually decreased by day 21 (Figure 4).

The fact of relative increase in iron concentration up to the control level after the sixth and seventh injections was established after the assessment of iron level in skeletal muscles of animals (except injection site). It was performed after the repeated injections with the preparation.

However, the characteristics of injection sites did not go through much change. Iron content in these tissues remained high through the entire research.

**Figure 5:**
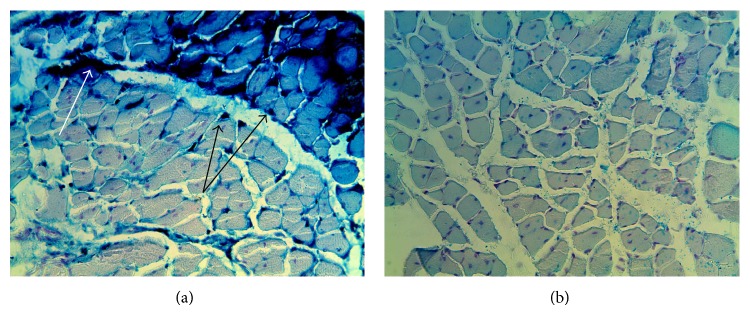
Site. Experiment. (a) Injection. Day 7 after the seventh injection with iron nanoparticles. (b) Area next to the injection site. Experiment. Day 7 after the seventh injection of iron nanoparticles. Magn. 300. Mainly exogenous iron is observed among the macrophages of endomysium and perimysium; it has dark blue staining, indicated with arrows (intensive Perls' reaction) (a).

In particular, the increased content of iron was observed in the muscle fibers and cell elements of endomysium in injection site on the seventh day after the fifth intramuscular injection (Figure 2). Muscle fibers and macrophages of connective tissue surrounding myosymplasts were filled with iron in the site of administration on the seventh day after the sixth injection with iron nanoparticles. The pictures of injection sites (Figure 5(a)) and adjacent tissues on day 7 after the seventh injection can be an illustration of that.

First of all, an increase of iron pool in body was accompanied by the increase of erythrocyte number and concentration of hemoglobin. In particular, the hemoglobin level in blood increased by 7.1% (*P* < 0.01) a day after the first injection. It increased by 11.3% (*P* < 0.05) after seven days.

**Figure 6:**
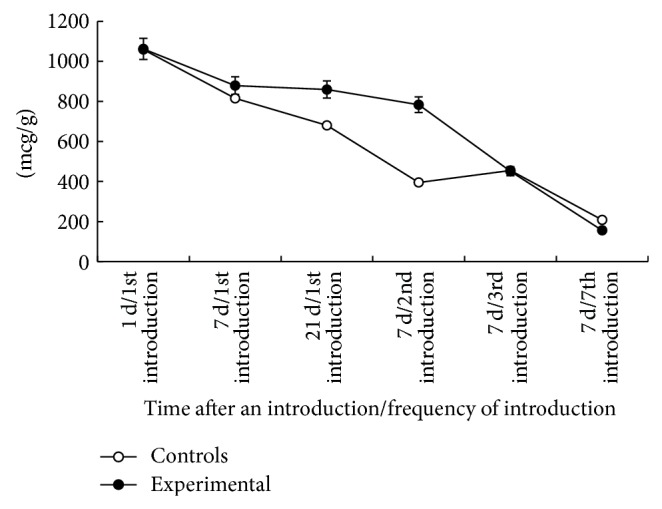
Concentration of iron in spleen after multiple intramuscular injections with iron nanoparticle agglomerates, mcg/g.

The concentration of iron in spleen of experimental rats changed likewise. Moreover, prior to the third injection, iron content in this organ was continuously increasing by 7.9% (*P* < 0.05) after seven days and by 26.3% (*P* < 0.001) by day 21 after the first injection. This difference attained maximum of 98.23% (*P* < 0.001) on the seventh day after the second injection; then the concentration of iron in the experimental group was continuously decreasing. It was assessed on the seventh day after the injection (Figure 6).

**Figure 7:**
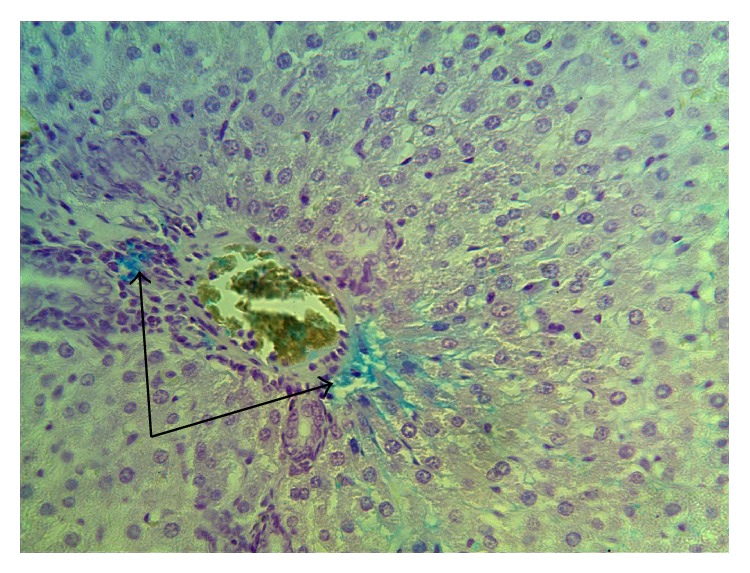
Liver. Experiment. The seventh day after the fifth injection of iron nanoparticles. Perls' positive reaction in macrophages of connective tissue. Magn. 300. Macrophage cells of connective tissue surrounding separate liver triads contain iron with blue staining (indicated with arrows).

**Figure 8:**
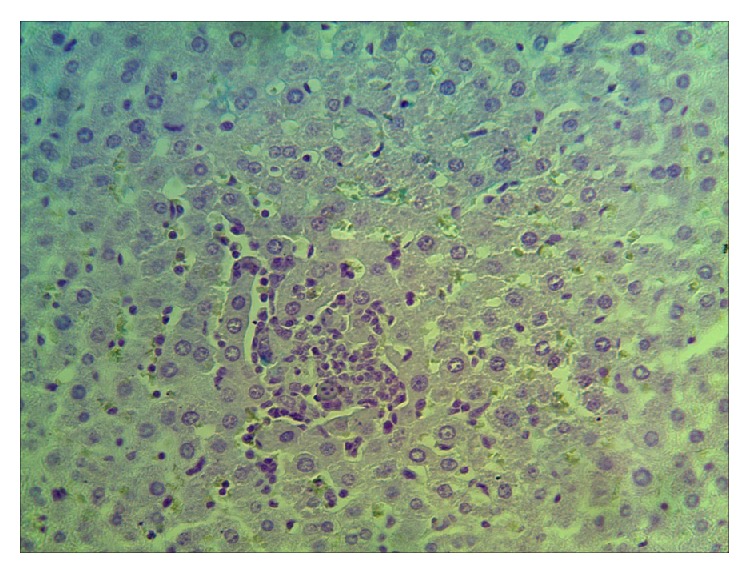
Liver. Experiment. The seventh day after the fifth injection of iron nanoparticles. Hematoxylin and eosin. Magn. 300. Perls' reaction. Proliferation of perisinusoidal macrophages inside separate liver lobules.

Single Kupffer cells were observed using Perls' reaction in the course of morphological studies of liver 14 and 21 days after a single administration of nanoparticle agglomerates. No positive reaction of Perls' for Kupffer cells was observed during the assessment of liver structure on the seventh day after 2, 3, 4, 5, and 6 injections. In the course of the study only single hepatocytes and Kupffer cells gave a positive reaction for the detection of iron in them. However, after five injections, lobular proliferation of Kupffer cells in separate lobules attested to the revitalization of these cells. At the same time, macrophage cells containing iron during the whole studied period were identified (Figure 7) in connective tissue surrounding certain liver triads. Local proliferation of perisinusoidal macrophages was observed in separate liver lobules (Figure 8).

It should be noted that multiple injections of iron agglomerates were not accompanied by any significant changes in morphological and functional characteristics of the studied organs. So, on the seventh day after the seventh intramuscular injection, liver of animals was characterized by the usual girder structure. No significant structural changes were found. Macrophages of connective tissue of the portal tracts and separate hepatocytes were characterized by Perls' positive reaction. The structure of the studied kidneys of experimental animals was not violated.

The expression of Caspase-3 during the whole experiment was 11 ± 0.08‰ in spleen of the control animals (Table 1).

On the seventh day after the injection, apoptosis of macrophages in the white pulp of spleen becomes active. It increased up to 2.1 ± 0.1‰ after the first introduction and up to 2.6 ± 0.5‰ (*P* < 0.01) after the third injection.

After the seventh injection, apoptosis becomes active in all structural areas of spleen (white and red pulp). The expression level was up to 2.9 ± 0.06‰ (*P* < 0.01). In addition, the overall number of macrophages in area unit decreased in this experimental point owing to their destruction by apoptosis. The reason is the increased apoptotic activity.

Analyzing the obtained data, we can assume that reduction of iron concentration in liver and muscle tissue of experimental animals during the first week after injection with preparation is a result of homeostasis stabilizing exchange of this element.

Differences in the intensity of iron elimination processes from nanoparticle preparation and its agglomerates can be illustrated by the data from the second experiment. In the first experiment, injections of nanoparticles are accompanied by a considerable increase of iron concentration in blood serum: on the first day by 22.2% (*P* < 0.01), and 12.2% (*P* < 0.01) on the seventh day after introduction (Table 2). Injections of agglomerates are followed by a slight increase of iron concentration in serum, only during the first days, by 4.3% (*P* < 0.01). Ferritin content after nanoparticle injections increased on the first day up to 27.6 ± 0.004 *μ*g/L and on the seventh day up to 31.8 ± 0.001 *μ*g/L, which was significantly higher than the value of the same indicator using the agglomerates. It was higher by 10.4 (*P* < 0.01) and 14.4% (*P* < 0.01), respectively.

Morphological composition of blood of experimental animals assessed according to the content of leukocytes and monocytes was consistently different. Content of these cells was significantly higher in blood of animals of group II than this level of the control group on the first and the seventh days, provided that for monocytes it was 1.8–2.1 times higher. At the same time, significant increase was observed for monocytes after introduction of nanoparticles only one day after the injection.

## 4. Discussion

Metal nanoparticles and their compounds have potential as microelement preparations; it was demonstrated in the researches [13–15]. At the same time, we know about the researches that demonstrate differences in biological characteristics of particles having different size [16–20].

In this regard, the assessment of microelement preparations containing nanoparticles with different size makes sense. Agglomerates are of interest too. Data obtained in our researches demonstrate relatively low permeability of iron from agglomerates (*d* = 431 ± 13.8 nm) as opposed to nanoparticle preparations (*d* = 80 ± 5 nm). So, the increase of iron concentration in blood serum was 22.2% on the first day after the injection with nanoparticles (experiment II), whereas introduction of agglomerates did not cause expressed changes in blood composition. The injected iron in the content of agglomerates was observed in the injection sites for a long term of more than 21 days. Long-term elimination of iron from injection sites shall be treated as the important positive attribute of nanoparticle agglomerate preparation.

In the research with nanoparticle agglomerates cell damage was not registered as a result of increasing free radical oxidation with excess iron content. It is well-known that in a number of reactions (Fenton, Haber-Weiss, Osipova) this element is able to lead to the formation of active forms of oxygen having a pathogenic effect on structural elements of the body.

Earlier studies [21] had shown the increase of apoptosis in liver and kidney cells of pigs at intramuscular injections of iron-containing preparation (iron dextran). In our researches, assessment of cells readiness to apoptosis using iron nanoparticle agglomerates in the studied organs (liver, spleen, and kidney) had revealed an expression of antibody Caspase-3 only in spleen.

Relatively slow release of iron with subsequent decrease in absorption level should be considered as an advantage of agglomerate preparation in terms of preventing the generation of nontransferrin bound Fe that can be observed following absorption of therapeutic doses of soluble iron [22–25]. At the same time, well defined action of nanoparticle agglomerates on content of many chemical elements in muscular tissue was observed in the course of our researches (Figure 3). If we consider these changes as a result of homeostatic body response, we can suppose that a small number of unbound nanoparticles in preparation could be the cause of this phenomenon.

Assessment of particle size in agglomerate preparation revealed the presence of 3–5% of particles sized 80 ± 5 nm; 9–11% of particles were 175 ± 12 nm. The results of our studies showed that a significant portion of iron particles with these sizes were in the bloodstream in the first hours after intramuscular injection. It is well known that expressed pathological effects of excess iron in body accompanied by the formation of free radicals and so forth are normally constrained by a well-functioning system of stabilization of its level [13]. Thus, the presence of iron particles in a small amount sized approximately 80–100 nm and more rapidly penetrating the blood within the first day invokes the mechanisms of homeostasis. It also leads to the decrease of iron content and many elements in tissues of animals. At the same time main part of preparation is localized in the injection site for more than 21 days.

This is also promoted by paramagnetic properties of iron nanoparticles and its oxides, determining additional formation of agglomerates in the muscle tissue.

Thus, there are different changes of iron metabolism in organism of animals caused by intramuscular injection of nanoparticles and their agglomerates. The duration of iron elimination from the depot formed by intramuscular injections allows considering preparation with iron nanoparticle agglomerates as a source of micronutrients in the treatment of elementosis.

## 5. Conclusions

Therefore, a morphological analysis of the injection site in experimental animals after repeated intramuscular introduction of iron nanoparticle agglomerates in dosage of 2 mg/kg of weight has shown that part of the iron injected is used at the injection site and is employed for myoglobin development; also it is captured by endomysium macrophages. The intensity of the reaction revealing iron at the injection site will increase mildly along with an increase in the number of injections.

## Figures and Tables

**Table 1:** The indexes of Caspase-3 antigen expression (∗) in spleen after an intramuscular introduction of iron nanoparticles in dosage of 2 mg/kg (in ‰).

Groups	Control	7 days after the first injection	7 days after the third injection	7 days after the seventh injection
	1.1 ± 0.08	2.1 ± 0.1	2.6 ± 0.05^**^	2.9 ± 0.06^**^

^*^Data are presented as mean (*X*) ± standard error of the mean (SE) (*n* = 1000); ^**^results are statistically significant (*P* < 0.01).

**Table 2:** Biochemical indicators in blood of rats in the period after injections of iron nanoparticles and their agglomerates.

Indicator	Group	Time after injection, days	
		1	7
Ferritin, *μ*g/L	1	27.6 ± 0.004	31.8 ± 0.001
	2	25.2 ± 0.003	28.9 ± 0.002
	3 (control)	26.9 ± 0.003	25.6 ± 0.002

Hemoglobin, g/L	1	165.4 ± 10.1	129.7 ± 9.1
	2	137.9 ± 14.9	122.3 ± 3.28
	3 (control)	126.7 ± 12.7	105 ± 5.5

Fe, mcmol/L	1	36.9 ± 0.004^**^	34.1 ± 0.001^**^
	2	31.5 ± 0.004^**^	30.3 ± 0.004
	3 (control)	30.2 ± 0.004	30.4 ± 0.009

Data are presented as mean (*X*) ± standard error of the mean (SE); ^**^results are statistically significant (*P* < 0.01).
